# Cardiovascular Imaging for Coronary Artery Disease in Patients with Diabetes Mellitus

**DOI:** 10.3390/jcm13133658

**Published:** 2024-06-23

**Authors:** Biljana Nedeljkovic Beleslin, Arif Al Nooryani, Branko Beleslin

**Affiliations:** 1 Clinic for Endocrinology, Diabetes and Metabolic Disorders, University Clinical Center of Serbia, 11000 Belgrade, Serbia; biljana_beleslin@yahoo.com; 2 Medical Faculty, University of Belgrade, Dr Subotica 8, 11000 Belgrade, Serbia; 3 Al Qassimi Hospital, Sharjah 3500, United Arab Emirates; arif345@yahoo.com; 4 Cardiology Clinic, University Clinical Center of Serbia, 11000 Belgrade, Serbia

**Keywords:** diabetes mellitus, non-invasive functional imaging, coronary flow reserve, fractional flow reserve, optical coherence tomography

## Abstract

In patients with diabetes mellitus, accelerated progression of atherosclerosis can lead to worse clinical outcomes. Determining the best diagnostic strategy to identify patients with increased cardiovascular risk is challenging. Current guidelines recommend using both functional imaging and CT angiography to detect myocardial ischemia and coronary artery disease based on pre-test probability. Functional imaging is suggested for patients with a higher clinical likelihood due to its higher rule-in diagnostic capacity. On the other hand, CT angiography is preferred for patients with lower pre-test probability because of its excellent negative predictive value. The optimal management strategy for asymptomatic diabetic patients remains unclear. In asymptomatic diabetic patients, previous randomized trials have not shown benefits from diagnostic testing over standard care. However, these trials were methodologically inconsistent and lacked clear stratification of cardiovascular risk. In terms of invasive evaluation, a combined invasive functional and anatomic imaging approach for angiographically intermediate coronary stenosis appears to be the best, most effective decision pathway for managing diabetic patients.

## 1. Introduction

With the increasing number of patients with diabetes mellitus and consequential risk of cardiovascular diseases (CVD) including coronary artery disease (CAD), stroke, heart failure, chronic renal disease, and atrial fibrillation, awareness of and the need for adequate and timely CV imaging are of paramount importance. The growing application of new glucose-lowering drugs—sodium/glucose-co-transporter-2 (SGLT2) inhibitors and glucagon-like peptuide-1 (GLP1) receptor agonists—has further promoted interest in the diagnosis of CVD in diabetic patients in order to prevent complications and adverse events as early as possible. Over the last two decades, we have witnessed exponential growth in the clinical presence of a variety of CV imaging modalities ranging from stress echocardiography and perfusion scintigraphy to cardiac magnetic resonance, CT angiography, sophisticated evaluation of coronary flow reserve via PET and invasive functional and morphological techniques.

When considering CV imaging in patients with diabetes, we can refer to several of the latest guidelines including the ESC guidelines on diabetes and CVD from 2019 [1] and 2023 [2], and 2019 ESC [3] and 2023 AHA/ACC [4] guidelines on chronic coronary syndrome/disease relevant to functional and anatomic CV imaging. There are also a number of relevant references in relation to CV imaging in patients with diabetes. In particular, we shall discuss different aspects and open issues of CV imaging and screening in asymptomatic diabetic patients both related and unrelated to the latest guidelines. 

## 2. Cardiovascular Imaging in Diabetic Patients: What do the Guidelines Recommend?

According to the 2019 ESC guidelines for diabetes, pre-diabetes, and cardiovascular disease [1], patients with diabetes are classified into very high (known CVD or target organ damage), high, and moderate cardiovascular risk. Most patients are at high CV risk with a duration of diabetes greater than 10 years, without target organ damage, or moderate risk including younger patients with a duration of diabetes of less than 10 years [1]. Regarding CV imaging, these guidelines [1] recommend only resting ECG in patients with additional hypertension and suspected CVD (Class I), whereas CT angiography or functional imaging (stress echocardiography, magnetic resonance imaging, or radionuclide perfusion imaging) may be considered in asymptomatic patients with diabetes for the screening of CAD (Class IIb). The question arises of the rationale of this diagnostic approach and why CV imaging has a limited recommendation level in asymptomatic patients with diabetes.

In fact, a meta-analysis of five randomized clinical trials [5] in 3299 asymptomatic patients with diabetes showed that non-invasive imaging including exercise stress ECG, stress echocardiography, myocardial perfusion imaging, and CT angiography did not affect clinical outcomes and reduce adverse events. Closer analysis of patients, methods, and strategy following testing disclosed a wide scatter of data, making a consistent conclusion quite difficult. The number of patients varied from 141 [6] to a maximum of 1123 in the DIAD trial [7], patients were not classified according to CV risk, and screening tests included various imaging modalities from low diagnostic profile exercise stress ECG to high diagnostic and prognostic capacity myocardial perfusion imaging, CT angiography, and coronary calcium score. In addition, the rate of positive screening tests ranged from 5.9% to 21.5% (corresponding to the overall rate of silent myocardial ischemia in diabetic patients), and the treatment strategy following testing was left to the discretion of the referring physician. Thus, the percentage of invasive coronary angiography procedures after positive non-invasive stress imaging ranged from 15% up to 93% [5]. Still, what was similar across different randomized trials was a very low annual event rate of 0.6–1.9% over 4–6 years of follow-up [5], and the absence of a significant effect of non-invasive screening on the occurrence of adverse cardiac events, except for the trial which had the lowest number of included patients and the highest number of invasive coronary angiographies following a positive test [6]. Therefore, differences in screening modality, further management, and revascularization strategy with low event rates may explain the lack of benefit for routine screening, leading to the recommendation that imaging functional testing and CT angiography may be recommended (IIb) [1] in high-risk asymptomatic subjects, particularly if other CV risk factors including peripheral artery disease, a high calcium score, or proteinuria are present. 

In general, these recommendations [1] correspond to the 2019 ESC guidelines for chronic coronary syndrome [3] which recommend non-invasive functional imaging or CT angiography in symptomatic patients based on pre-test probability and clinical likelihood of CAD, patient characteristics, local availability, practice, and expertise. Patients with lower pre-test probability are referred to CT angiography while patients with a higher likelihood of having CAD are directed to functional imaging. In the case of very high clinical likelihood, typical symptoms at a low level of physical activity refractory to medical therapy, and a high risk of adverse events, invasive angiography is recommended without prior non-invasive testing. 

Surprisingly, the latest 2023 ESC guidelines on the management of CVD in diabetes [2] did not introduce new information to the diagnostic strategy or screening for asymptomatic diabetic patients. Instead, it entirely refers to CV imaging in the previous 2019 ESC guidelines on diabetes, pre-diabetes, and cardiovascular diseases [1]. The only recent addition to the list of screening randomized trials is the DANCAVAS trial, which again did not show a benefit of routine CVD screening on any cause of death over 5 years [8].

Therefore, the non-invasive diagnostic strategy for patients with diabetes and without established atherosclerotic CVD remains challenging according to the current guidelines [1,2]. Functional imaging stress testing and CT angiography may be considered and indicated in patients with diabetes. 

## 3. Coronary Artery Calcium Scoring and CT Angiography

Of particular prognostic value appears to be the coronary artery calcium (CAC) score determined using CT, where each increment (Agatston score 0–99, 100–399, >400) is associated with increased mortality [9]. Therefore, the CAC score determined using CT may be considered a risk modifier in the CV risk assessment of asymptomatic patients with diabetes [1]. Valensi et al. [10] also demonstrated that in high-risk asymptomatic diabetic patients determined to have target organ damage or several risk factors, the most effective strategy to detect coronary stenosis eligible for revascularization is to perform myocardial scintigraphy in patients with severe target organ damage or CAC score (≥100 Agatston units) in patients without severe target organ damage but several risk factors. This is in line with the PROMISE [11] and SCOT-HEART trials [12], which changed clinical practice and guidelines by introducing for the first time CT angiography equivalent to functional CV imaging [3]. The PROMISE randomized trial [11] in more than 10,000 patients did not show a significant difference in clinical outcomes between the initial CT angiography strategy and functional testing. The SCOT-HEART trial [12] demonstrated a significantly lower rate of cardiac death and non-fatal myocardial infarction over 5 years when CT coronary angiography was performed in addition to standard care testing, without more coronary angiography or coronary revascularization [12]. In addition, in a post hoc analysis of the SCOT-HEART randomized study, Williams MC et al. [13] showed that low attenuation non-calcified plaque burden, as detected using CT angiography, was the strongest predictor of future adverse events with almost five times higher likelihood of subsequent myocardial infarction.

However, the FACTOR-64 randomized trial [14], comparing CT coronary angiography with optimal guideline-directed medical therapy, showed that among asymptomatic patients with diabetes, only a minority (<25%) were found to have moderate or severe coronary atherosclerosis. Changes in medical care, instituted as a result of the CT angiography screening for CAD, did not reduce the rate of all-cause mortality, non-fatal MI, or unstable angina requiring hospitalization. The overall event rate was lower than expected, and only 6% were found to have CAD severe enough to justify coronary revascularization. Still, CT angiography holds a high negative predictive value for excluding CAD with high accuracy in individuals with lower clinical likelihood [15] of CAD. Regarding CT angiography-based novel techniques, we should mention the emerging role of CT in combining anatomical information with functional hemodynamic assessment, which includes fractional flow reserve derived from CT (FFRCT) and perfusion CT [16].

## 4. Silent Myocardial Ischemia and Myocardial Infarction in Diabetic Patients

One-fourth to one-third of patients with acute coronary syndrome have diabetes, and screening for diabetes is recommended in all patients with CVD, especially acute coronary syndrome [2]. On the other hand, it is recommended to assess clinical symptoms suggestive of atherosclerotic CVD or CAD in all patients with diabetes [2]. However, a considerable proportion of patients with diabetes have silent myocardial ischemia or even previous asymptomatic myocardial infarction. In patients with diabetes, silent myocardial ischemia (ST-segment depression) on exercise ECG is present in 20–40% of patients [17], and the diagnostic value of exercise ECG is moderate [18]. However, asymptomatic diabetic patients have more ischemia upon myocardial perfusion scintigraphy than upon exercise [19]. Additionally, myocardial perfusion scintigraphy with robust sensitivity has demonstrated that in asymptomatic diabetic patients, post-stress left ventricular ejection fraction and stress-induced ischemia are independent predictors of CAD mortality and myocardial infarction [20]. Unrecognized myocardial infarction carries not only a worse prognosis in symptomatic diabetic patients but also a worse prognosis in asymptomatic ones. In fact, unrecognized myocardial infarction was present in almost 20% of asymptomatic diabetic patients (compared to 5% by ECG) as detected via delayed enhanced cardiac magnetic imaging [19]. Most importantly, unrecognized myocardial infarction was associated with a significantly higher rate of death and myocardial infarction in a 5-year follow-up period [21]. Also, diffuse myocardial fibrosis determined using cardiac magnetic resonance imaging carries a worse prognosis and clinical outcome [22]. Intramyocardial fatty scars, most likely related to previous silent myocardial infarction, can be detected using CAC CT angiography more often in heavily calcified patients (40%) but also in patients with a CAC score of 0 (12%) [23]. The prognostic value of these findings, particularly in non-calcified coronary arteries, needs to be determined. 

## 5. Stress Echocardiography and Coronary Flow Reserve

Regarding the role of resting echocardiography in patients with diabetes, it has been shown that patients with diastolic dysfunction and increased left ventricle mass [24,25] have poorer clinical outcomes. 

Earlier studies have demonstrated that the outcome of patients with diabetes and myocardial ischemia on stress echocardiography testing is significantly worse than in non-diabetic patients [26]. In addition, reduced (≤2.0) coronary flow reserve (CFR) determined using 2D echocardiography in patients with diabetes and negative dipyridamole stress echocardiography carries highly significant and convincing worse outcome in comparison with preserved CFR (>2.0) over a follow-up of 3 years (58% vs. 16%, *p* < 0.001) [27]. In addition, Murthy VL et al. [28] demonstrated that abnormal CFR (≤1.6) determined using PET in patients with diabetes and without established coronary artery disease has a significantly higher annualized rate of cardiac mortality in comparison to preserved CFR (>1.6) and is similar to patients with diabetes and established coronary artery disease (2.8% vs. 0.3% vs. 2.9%, respectively). We have also previously demonstrated that the outcome of diabetic patients with abnormal CFR determined using echocardiography and CAC of >200 is significantly worse than in patients with preserved CFR [29]. Thus, positive findings on stress echocardiography testing and particularly reduced coronary flow reserve determined using echocardiography or PET are independent predictors of poor outcomes in patients with diabetes. In addition, a strategy based on stress echocardiography (exercise or pharmacological) is cost effective and carries no risk of radiation exposure compared to radiation-based modalities (angiography, SPECT, and PET) in low-risk chest pain patients [30,31]. There is not only an immediate diagnostic cost-effective benefit but also a reduced projected risk of cancer over time [31].

## 6. Invasive Functional Assessment

Invasive functional assessment either with fractional flow reserve (FFR) or other non-hyperemic pressure ratios (e.g., iFR) is at the center of the invasive diagnostic algorithm and should be performed in angiographically intermediate coronary stenoses before proceeding with percutaneous coronary intervention [4,32]. FFR or other non-hyperemic invasive functional indices lower the risk of hard adverse cardiac events [33] and mortality [34]. According to the latest AHA/ACC guidelines for the management of patients with chronic coronary disease [4], in patients with chronic coronary disease undergoing coronary angiography without prior stress testing, the use of FFR to evaluate angiographically intermediate coronary stenosis is considered an intervention of high economic value [4]. Therefore, FFR is not only a clinically useful invasive method but also cost-effective. Nowadays, the historical term of “significant” coronary stenosis on angiography has been replaced by angiographically intermediate (40–90%) coronary stenosis which should be tested using invasive functional evaluation in the absence of previous non-invasive testing. Only high-grade (≥90%) coronary stenosis is considered critical and should be revascularized without further invasive testing if associated with obvious symptoms and signs of myocardial ischemia [4,32]. However, in certain clinical conditions, such as patients with diabetes, the value of FFR is not as well validated and established. A study by Kennedy M.W. et al. [35] showed that deferred revascularization based on FFR in 250 patients was associated with worse outcomes in diabetic patients compared to non-diabetic patients, with significantly more target lesion revascularizations (16% vs. 6%) during a mean follow-up of 40 months. While earlier large multicenter studies [36,37] did not show significant differences in outcomes between diabetic and non-diabetic patients based on FFR values, this issue remains controversial. The recent COMBINE OCT FFR [38] trial investigated the impact of both functional (FFR) and intravascular morphologic characteristics as analyzed using optical coherence tomography (OCT)-detected thin-cap fibroatheroma (TCFA) on the clinical outcomes of patients with diabetes and angiographically intermediate coronary stenosis (40–80% coronary stenosis determined by visual assessment). TCFA, considered a vulnerable atherosclerotic plaque, is defined as a predominantly lipid-rich plaque with the thinnest part of the atheroma cap measuring ≤ 65 μm via OCT evaluation. The study found that among 550 enrolled diabetic patients with ≥1 FFR-negative angiographically intermediate lesions, TCFA, or an atherosclerotic vulnerable plaque, was present in 25% of the patients [38]. The presence of TCFA findings was associated with a significantly higher rate (13.3% vs. 3.1%, *p* < 0.001) of adverse coronary events (composite of cardiac death, target vessel myocardial infarction, target lesion revascularization, and hospitalization due to unstable or progressive angina) despite a preserved FFR value of >0.80. These findings highlight the impact of both plaque vulnerability and ischemia on future adverse events and may explain the worse outcomes in diabetic patients with negative FFR. In other words, the progression of atherosclerosis in diabetic patients differs from that in patients without diabetes. However, the best strategy to manage vulnerable plaques in angiographically intermediate coronary lesions with preserved FFR remains a question due to low hard event rates, as well as the effect of lipid-lowering therapy on the regression of coronary plaque volume and increases in fibroatheroma cap thickness [39,40]. 

Microvascular disease is common among diabetic patients, often preceding epicardial atherosclerosis and diabetic cardiomyopathy. In fact, invasive assessment of microcirculatory dysfunction is one of the fast-growing areas in invasive cardiology with the development of new functional indices. Invasive evaluation of FFR, CFR, and the index of microcirculatory resistance derived from bolus thermodilution are considered standard parameters to diagnose microcirculatory dysfunction [41]. Recently, the principle of continuous thermodilution was introduced to measure absolute flow and myocardial resistance reserve (MRR) independently of autoregulation and myocardial mass [42]. It has been shown [42] that in the diabetic cohort, CFR and MRR were significantly lower compared to the non-diabetic group (CFR = 2.38 ± 0.61 and 2.88 ± 0.82; MRR = 2.79 ± 0.87 and 3.48 ± 1.02 for diabetic and non-diabetic patients respectively). Interestingly, in the same group of patients, it has been shown that left atrial reservoir strain, an early marker of diastolic dysfunction, significantly deteriorated, confirming the link between coronary microcirculatory dysfunction and cardiomyopathy in patients with diabetes [43].

## 7. New Cardiovascular Risk Stratification in Patients with Diabetes 

The latest 2023 ESC guidelines for the management of CVD in patients with diabetes [2] introduce a new risk score, SCORE2-Diabetes, which corresponds to the SCORE2 CV risk score from the latest CVD prevention guidelines [44]. For patients with type 2 diabetes mellitus without symptomatic atherosclerotic CVD or severe target organ damage, it is recommended to estimate the 10-year CV risk score using the SCORE2-Diabetes algorithm [2]. SCORE2-Diabetes combines information on CV risk factors with diabetes-specific information and is calibrated for four clusters of European countries with low, moderate, high, and very high risk. This risk stratification for different European regions is valuable because the CV risk in diabetic patients may be 2–3 times higher in very high-risk regions, mostly eastern European countries compared to low-CV-risk regions predominantly in northern and western European countries [2]. Specifically, SCORE2-Diabetes categorizes asymptomatic diabetic patients aged 40 and above into very high risk (≥20% estimated 10-year CVD risk, clinically established atherosclerotic CVD and severe target organ damage), high risk (10 to <20% 10-year CVD risk, without established atherosclerotic CVD or severe target organ damage), moderate CV risk (5 to <10% 10-year CVD risk, without established atherosclerotic CVD or severe target organ damage), and low CV risk (<5% 10-year CVD risk, without established atherosclerotic CVD or severe target organ damage) [2]. 

## 8. Conclusions

The proportion of patients with type 2 diabetes mellitus and prediabetes is increasing worldwide with specific regions such as Middle Eastern countries already having more than 50% of patients with atherosclerotic CVD also being diabetic. The new SCORE2-Diabetes risk score [2] is important as it sets a practical and scientific framework for screening asymptomatic patients according to risk category which was not the case in earlier studies. 

**Figure 1:**
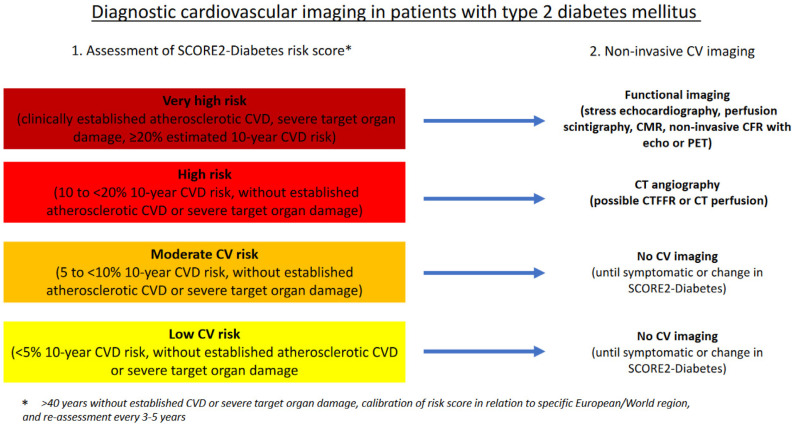
Diagnostic cardiovascular imaging in patients with type 2 diabetes mellitus.

How can we interpret the data from different studies on CV imaging in diabetes and the guidelines for diabetes that do not recommend systematic screening in asymptomatic subjects [1,2,45]? Symptomatic diabetic patients are considered high or very high CV risk patients and have a high probability of having CAD. It is preferable for them to undergo CV functional imaging testing because these tests have a greater capacity to identify patients with significant CAD [46]. The diagnostic management of asymptomatic diabetic patients is more debatable, and nowadays, the best strategy is likely based on the assessment of the SCORE2-Diabetes score. Patients with high CV risk should preferably be referred for screening with CT angiography if their characteristics allow for non-invasive anatomic testing with CT (CAC scoring as a first step), while patients with low-to-moderate CV risk probably do not need CV screening unless they have multiple CV risk factors (Figure 1). It is important to consider that any CV risk score is a variable parameter and is influenced over time by age, preventive measures, and CV drugs. Therefore, contemporary preventive measures and lifestyle changes recommended by doctors and health authorities, particularly for diabetic patients, may be more effective than functional and anatomic screening for CVD. Even sophisticated and diagnostically powerful CV imaging methods such as CT angiography to detect subclinical atherosclerosis or perfusion imaging to detect silent myocardial ischemia may fail to identify high-risk asymptomatic patients. Finally, in cases where invasive evaluation is necessary due to myocardial ischemia or the uncertainty of previous non-invasive test results, both functional and intravascular imaging data should be considered for the best diagnostic work-up and patient management. Further trials based on risk stratification are needed to establish the best diagnostic strategy for assessing CVD in patients with diabetes mellitus.
